# Design, Synthesis, and In Vitro Activity of Pyrazine Compounds

**DOI:** 10.3390/molecules24234389

**Published:** 2019-12-01

**Authors:** Panagiotis Parsonidis, Mahammad Shaik, Athanasia Panagiota Serafeim, Ioanna Vlachou, Vasiliki Daikopoulou, Ioannis Papasotiriou

**Affiliations:** 1 RGCC S.A. Industrial Area of Florina, GR53100 Florina, Greece; parsonidis.panagiotis@rgcc-genlab.com (P.P.); athanasia.serafeim@rgcc-genlab.com (A.P.S.); vlachou.ioanna@rgcc-genlab.com (I.V.); daikopoulou.vasiliki@rgcc-genlab.com (V.D.); 2 Medicinal Chemistry Division, Rgcc India Private Limited, Hyderabad 500090, India; shaik.mahammad@rgcc-india.com; 3 RGCC International GmbH, Baarerstrasse, 956301 Zug, Switzerland

**Keywords:** structure-based drug design, pyrazine compounds, protein tyrosine phosphatases

## Abstract

Despite the fact that there are several anticancer drugs available, cancer has evolved using different pathways inside the cell. The protein tyrosine phosphatases pathway is responsible for monitoring cell proliferation, diversity, migration, and metabolism. More specifically, the SHP2 protein, which is a member of the PTPs family, is closely related to cancer. In our efforts, with the aid of a structure-based drug design, we optimized the known inhibitor SHP099 by introducing 1-(methylsulfonyl)-4-prolylpiperazine as a linker. We designed and synthesized three pyrazine-based small molecules. We started with prolines as cyclic amines, confirming that our structures had the same interactions with those already existing in the literature, and, here, we report one new hydrogen bond. These studies concluded in the discovery of methyl (6-amino-5-(2,3-dichlorophenyl)pyrazin-2-yl)prolylprolinate hydrochloride as one of the final compounds which is an active and acceptable cytotoxic agent.

## 1. Introduction

The definition of cancer is that it is a disease caused by an uncontrolled division of abnormal cells in a part of the body. According to the World Health Organization [1], 9.6 million people worldwide are estimated to have died from cancer in 2018, making cancer the second most deadly disease globally. The most common cancers are lung (2.09 million cases), breast (2.09 million cases), and colorectal (1.8 million cases). Thus, discovery of novel drugs for the treatment of cancer patients is vital.

The activation of the PTP’s (Protein tyrosine phosphatases) pathway in human cancers has triggered the development of a variety of pharmacological inhibitors targeting catalytic sites of the SHP2 cascade. The SHP2 protein belongs to the protein tyrosine phosphatases group of enzymes. It is a non-receptor protein which is constituted from two SH2 (N-terminal Src homology 2) domains, a PTP domain, and a C-terminal tail. The activity of SHP2 is auto-inhibited, as the N-SH2 domain is binding with the PTP domain [2]. The upregulation expression of the *PTPN11* gene that encodes SHP2 protein exists in melanoma [3,4], liver cancer [5,6], and lung cancer [7].

**Figure 1:**
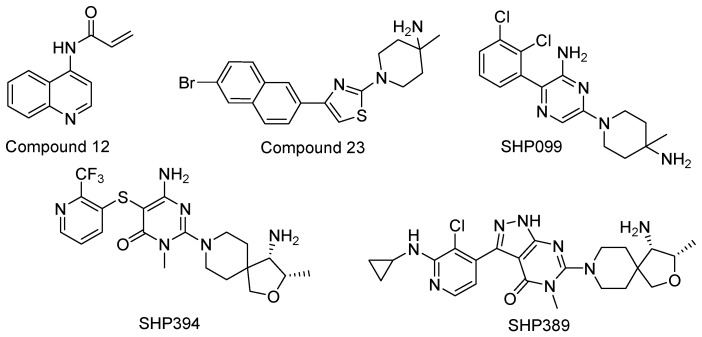
The Tyrosine phosphatase SHP2 allosteric inhibitors according to the literature.

More specifically, the SHP2 oncogene activates the RAS-ERK signaling pathway, so it can regulate cancer cell survival and proliferation [8,9]. In 2016, Fortanet et al. [10] revealed a new allosteric binding site of SHP2 with X-ray analysis. After structure-based drug design, they synthesized a small molecule, SHP099. The same molecule was used by Chen’s group [11], who reported that receptor tyrosine kinases-driven cancer cells depend on SHP2 survival. One year later, LaRochelle et al. [12] followed an alternative route to find allosteric inhibitors by making use of a partially active cancer mutant, SHP2F285S. In recent years, many different attempts have been performed for the discovery of new SHP2 inhibitors [13]. Cyanoacrylamides [14], 6-amino-3-methylpyrimidinones [15], substituted thiazoles [16], and fused bicyclic compounds [17] have shown interesting results in SHP2 inhibition (Figure 1).

In order to evaluate and determine the utility of substituted pyrazine compounds, such as SHP2 inhibitors, we built a pharmacophore model using the highly selective SHP2 allosteric inhibitor SHP099-SHP2 complex. In the present work, we designed three small molecules using structure-based drug design. The substituted pyrazine compounds incorporated with novel 1-(methyl sulfonyl)-4-prolylpiperazine, as a linker to amides and sulphonamides, and the cytotoxic effect on different human cancer cell lines were examined.

## 2. Results

### 2.1. Structure Retrieval and Validation

**Figure 2:**
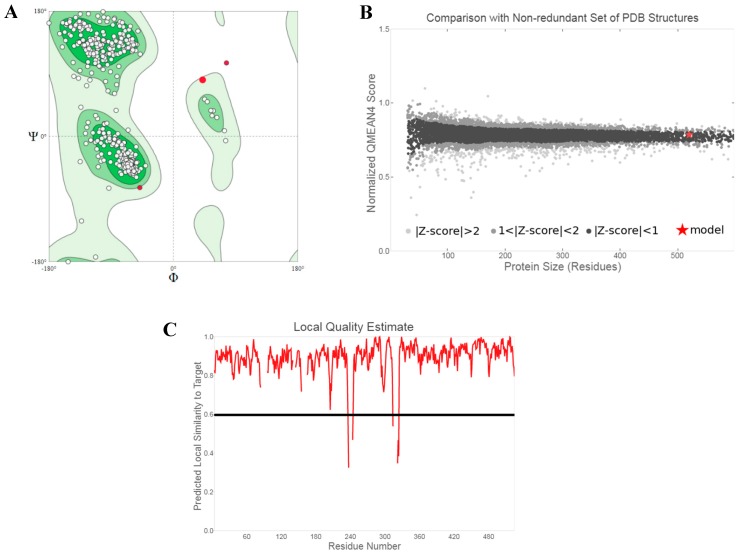
(**A**) Ramachandran plot analysis to assess the chemical correctness of backbone φ and ψ angles of SHP2 structure 5EHR. (**B**) QMEAN4-based comparison plot. The structure of 5EHR is indicated using a red star. (**C**) QMEANDisCo-based local quality plot. The threshold of 0.6 is indicated by a black line below which low-quality residues are found.

The three-dimensional structure of SHP2 was downloaded from the Research Collaboratory for Structural Bioinformatics (RCSB) Protein Data Bank and used for the structure-guided design of low molecular weight organic compounds [18]. The crystal structure (PDB ID: 5EHR) was solved through X-ray crystallography with a resolution of 1.7 Å and contained 526 amino acids which covered 87.8% of the canonical protein sequence (UniProtKB ID: Q06124) [11]. The quality of the crystal structure was validated using the SWISS-MODEL Structure Assessment tools [19]. Ramachandran plot analysis showed that 96.62% of the residues were within energetically favored regions, while only 0.85% (VAL505, LYS324, GLU313, GLY115) were Ramachandran outliers (Figure 2A) [20]. The outliers were found adjacent to the gaps in the protein structure. The Qualitative Model Energy Analysis 4 (QMEAN4) global score of 5EHR indicates that the overall quality of the structure was good when compared to experimental structures of similar size (Figure 2B) [21]. Assessment of the local quality of the structure showed that only a few compounds were characterized by low quality (Figure 2C).

The allosteric inhibitor SHP099 was co-crystallized with the protein structure of SHP2. The SHP099 (Chemical name: 6-(4-azanyl-4-methyl-piperidin-1-Yl)-3-(2,3-bis(chloranyl)phenyl)pyrazin-2-amine) is a highly potent and selective allosteric inhibitor of SHP2 with an IC_50_ value of 0.071 µM. The inhibitor binds to the interface of the N-terminal SH2, C-terminal SH2, and PTP domains and stabilizes an auto-inhibited conformation of SHP2 [11]. We used the above protein structure for the structured-guided design of novel allosteric SHP2 inhibitors.

### 2.2. Small Molecule Docking

**Figure 3:**
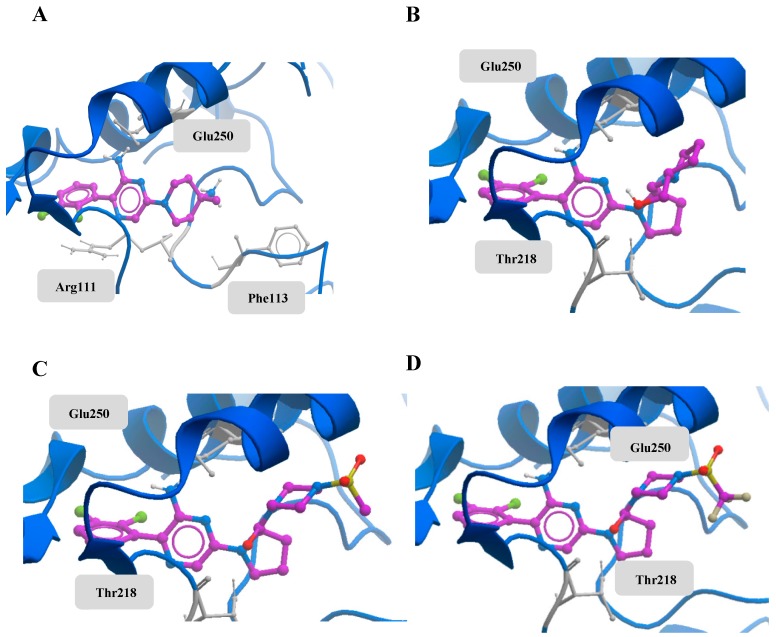
(**A**) Docked conformation of SHP099, (**B**) docked conformation of compound **10**, (**C**) docked conformation of compound **18**, and (**D**) docked conformation of compound **19**. The residues in grey form hydrogen bonds with the compound.

**Scheme 1:**
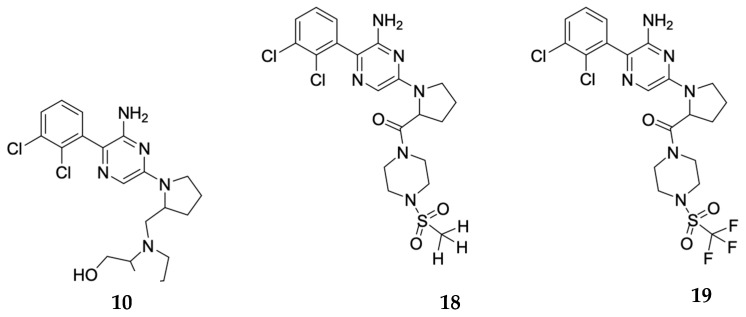
Compounds after docking evaluation.

An in silico study based on molecular docking was implemented to explore the interactions between the newly synthesized small molecules and the allosteric binding site on SHP2. Docking was performed against the 3D structure of SHP2, and the docking scores and binding poses of the compounds were obtained. Based on a co-crystallized ligand, SHP099, several compounds were designed in order to improve the binding affinity. To enhance the affinity between the binding pocket and the compounds, the third ring was changed while the aminopyrazine moiety and the dichlorophenyl moiety were kept identical to the reference compound. That design path was followed, because the two last moieties had the strongest bonds in the binding pocket, while the third part could be improved. Compounds **10**, **18**, and **19** (Scheme 1) showed scores comparable to that of the co-crystallized ligand, SHP099. The models of the protein–ligand complexes reveal the interactions between compounds **10**, **18**, and **19** and the surrounding residues of the binding pocket (Figure 3). All three compounds form a hydrogen bond with Glu250 of SHP2′s PTP domain and a hydrogen bond with Thr218 that lies on the loop between domains C-SH2 and PTP. Hydrophobic interactions between the dichlorophenyl ring and residues of the PTP domain are shared with Thr218 which lies on the loop between domains C-SH2 and PTP. Hydrophobic interactions between the dichlorophenyl ring and the surrounding residues further aid the protein–ligand interactions.

### 2.3. Synthetic Strategy

**Scheme 2:**
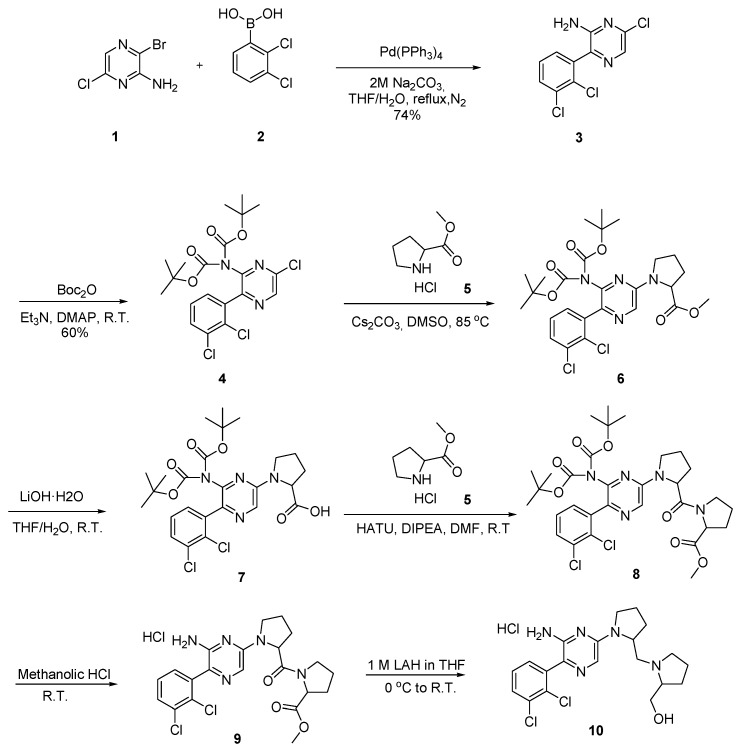
Synthesis of compound **10**.

The synthesis of compound **10** (Scheme 2) started with the Suzuki–Miyaura coupling between (2,3-dichlorophenyl) boronic acid (**2**) and 3-bromo-6-chloropyrazin-2-amine (**1**), affording intermediate (**3**) in 74% yield, and then protection of amine on pyrazine with di-tert-butyl dicarbonate gave the di-protected intermediate (**4**) in 60% yield. Then, a S_N_Ar reaction on this biphenyl intermediate with l-proline methyl ester (**5**) provided the proline ester intermediate (**6**) with 47% yield. Ester hydrolysis with base in homolytic mixture of water and THF (tetrahydrofuran) ended up with acid intermediate (**7**) in 54% yield. Amide coupling with l-proline methyl ester (**5**) resulted in proline amide intermediate (**8**) with 49% yield. Final deprotection using 4 M HCl in MeOH gave a 36% yield of compound **9**, which, upon reduction with LAH (lithium aluminum hydride) in THF, afforded compound **10** as free base after acid/base extraction with very low yield (13%).

**Scheme 3:**
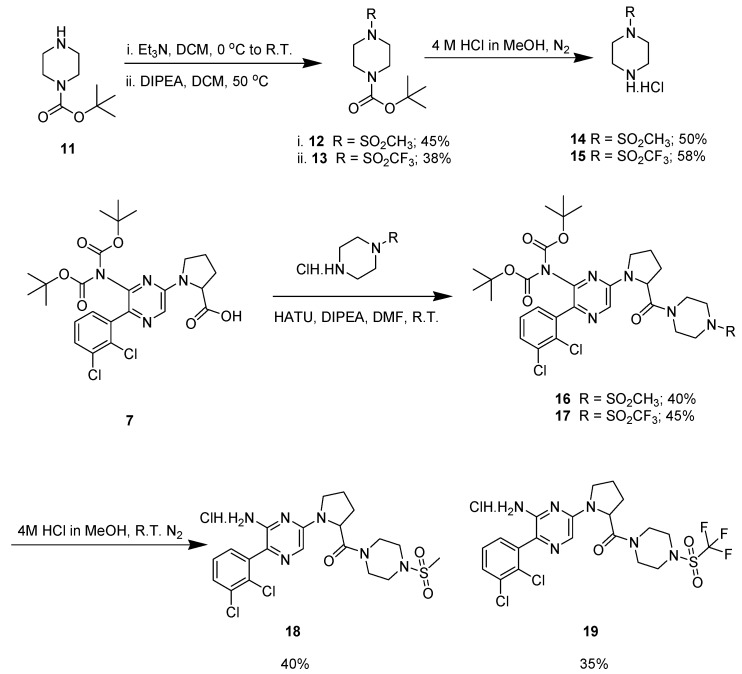
Synthesis of compounds **18** and **19**.

The synthesis of compounds **18** and **19** (Scheme 3) started with the sulphonamide synthesis on tert-butyl piperazine-1-carboxylate (**11**) with methane sulfonyl chloride and trifluoromethane sulfonic anhydride and ended up with intermediates **12** and **13**. Deprotection with 4 M methanolic HCl resulted in intermediates **14** and **15** with 50% and 58% yields, respectively. Amide coupling between (6-((ditert-butoxycarbonyl)amino)-5-(2,3-dichlorophenyl)pyrazin-2-yl)proline (**7**) and intermediates **14** and **15** resulted in intermediates **16** and **17** in 40% and 45% yields, accordingly. Final deprotection of amine on pyrazine gave the final compound **18** with a 40% yield and compound **19** with a 35% yield.

**Scheme 4:**
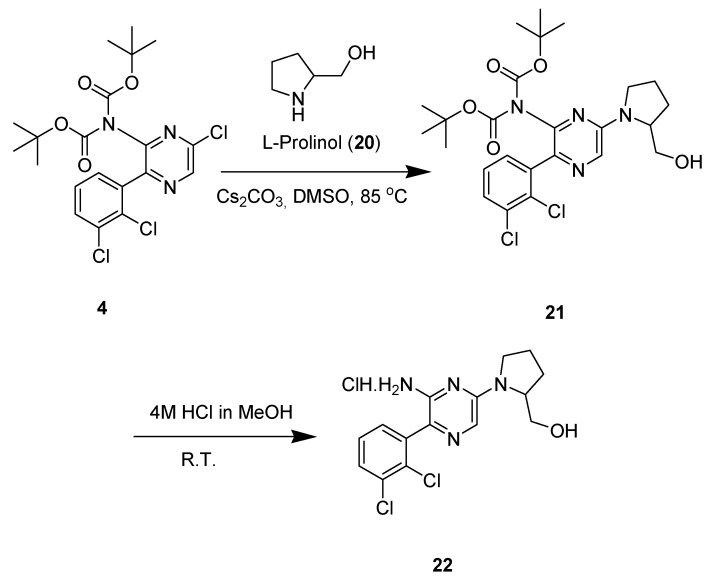
Synthesis of intermediates **21** and **22**.

The synthesis of compound 10 was performed by another synthetic route in order to increase the final yield. We succeeded to synthesize two different intermediates which were tested for their biological activity. However, we did not receive the final product (compound **10**) with this synthetic route. The synthesis of **5** and **6** (Scheme 4) started with an S_N_Ar reaction on the Boc protected biphenyl intermediate (**4**) with l-prolinol (**17**) and which provided the pyrazine ortho prolinol compound **5** with a 60% yield. Compound **5** on deprotection with 4 M methanolic in MeOH gave compound **6** as yellow colored hydrochloride salt with a 45% yield.

### 2.4. Biological Effect of Intermediates (**21**, **22**) in Human Cancer Cell Lines

Cytotoxic effect of the first two intermediates on MCF7, MDA-MB-231, HCT116, and MOR was evaluated using the MTT assay. Time- (24, 48, 72 h) and dosage- (0.001–100 µM) dependent inhibition of the viability of cancer cells was estimated. According to our results, at 48 h, MCF7 showed a statistically significant decrease in viability at 0.1 µM (30%) and at 0.01 µM (42%) of intermediate **21**. At 48 h, there was a significant effect on HCT116 viability with 0.1 µM (17%) and 0.01 µM (16%) of intermediate **21**. Intermediate **21** did not have any statistically significant inhibition in cell proliferation of MDA-MB-231 and MOR at all time points. There was no statistically significant result on MCF7 after treatment with intermediate **22** at all time points. Human breast adenocarcinoma cell line MDA-MB-231 appeared to have significantly reduced viability at 72 h with 1 µM (20%) and 0.1 µM (15%) of intermediate **22**. There was a significant effect on HCT116 viability at 48 h with 1 µM (23%) and at 72 h with 0.1 µM (31%) of intermediate **22**. The significant effect on MOR cell proliferation observed at 24 h with 0.1 µM (25%) and with 0.01 µM (23%) of intermediate **22**.

### 2.5. Biological Effect of Intermediate **9** and final **10** in Human Cancer Cell Lines

Intermediate **9** and final **10** compoundswere tested with MTT, SRB, and CVE assays to determine the effect they might have on the four human cancer cell lines. Intermediate **9** (Table 1) had a significant effect on MCF7 proliferation at 24 h with 0.1 µM (17%) with CVE, at 48 h with **0.001 µM** (27%) with MTT and with **0.001 µM** (9%) with CVE, and at 72 h with 0.001 µM (12%), 0.01 µM (11%), and **1 µM** (9%) MTT and with 0.1 µM (7%) and **1 µM** (10%) SRB. The two concentrations that are highlighted in **bold** (i.e., 0.001 and 1 µM) showed significant effects at the same time point at two of the three assays. There was no significant effect of intermediate **9** on MDA-MB-231 at all time points and all assays. Human colon cancer cell line HCT116 showed significant reduced viability after the treatment with intermediate **9** only at the MTT assay at 24 h with 0.01 µM (18%) and 0.1 µM (16%). Intermediate **9** significantly decreased the viability of MOR at 24 h with 0.001 µM (20%) and 0.1 µM (13%) MTT, at 48 h with 0.001 µM (19%), **0.01 µM** (16%) MTT, and **0.01 µM** (13%) SRB, and at 72 h with 0.001 µM (30%), **0.01 µM** (31%), **0.1 µM** (18%) MTT, with **0.1 µM** (9%) CVE and with **0.01 µM** (25%) SRB. Similarly, the concentrations highlighted in **bold** (i.e., 0.01 and 0.1 µM) showed significant effects at the same time point for two of the three assays.

Final compound **10** (Table 2) demonstrated a significant effect on MCF7 viability at 24 h with 0.001 µM (13%) and 0.1 µM (20%) MTT and 1 µM (10%) CVE, at 48 h with 0.01 µM (6%) CVE, and at 72 h with 0.001 µM (8%), 0.01 µM (11%), 0.1 µM (15%), and 1 µM (8%) MTT. The effect on MDA-MB-231 viability was observed at 24 h with 0.01 µM (16%) and 0.1 µM (30%) MTT and at 72 h with 0.1 µM (13%) MTT. The MTT was the only assay that gave statistically significant results on HCT116 proliferation at 24 h with 0.001 µM (19%), 0.01 µM (14%), 0.1 µM (32%) and at 72 h with 0.01 µM (25%) and 0.1 µM (22%). No significant effect with the CVE assay was observed on MOR viability. The MTT showed significant effects at 24 h with 0.001 µM (17%), 0.01 µM (28%), and 0.1 µM (27%) and at 48 h with 0.01 µM (31%) and 0.1 µM (23%), and at 72 h with 0.001 µM (25%), 0.01 µM (38%), and 0.1 µM (33%). With the SRB assay, the only significant effect was at 48 h with 1 µM (24%).

Intermediate **9** and final molecule **10** were also assessed for their biological activity on the four human cancer cell lines with flow cytometry by Annexin V and 7-AAD double staining. Annexin V positive cells indicated early apoptotic cells, 7-AAD positive cells indicated necrotic cells, double positive cells represented the late apoptotic cells, and the double negative cells indicated the living cells. The concentrations that appeared (Table 3) to have a significant effect on MCF7 cell viability at the same time point in two of the three assays were at 48 h with 0.001 µM and at 72 h with 1 µM of intermediate **9**. The percentages of untreated cells were 88.48% for living cells, 0.41% for early apoptotic, 1.84% for late apoptotic, and 1.3% for dead cells. After treatment with 0.001 µM of intermediate **9,** the percentages of cells were 86.53% for living cells, 0.38% for early apoptotic, 1.38% for late apoptotic, and 2.34% for dead cells. Generally, an increasing dose of intermediate **9** led to an increasing percentage of apoptosis and necrosis. At 72 h, the percentages of untreated cells were 78.21% for living cells, 1.62% for early apoptotic, 6.45% for late apoptotic, and 1.52% for dead cells. After treatment with 1 µM of intermediate **9**, the percentages of cells were 78.21% for living cells, 1.51% for early apoptotic, 5.91% for late apoptotic, and 2.34% for dead cells.

Similarly, the concentrations that appeared (Table 4) to have a significant effect on MOR cell viability at the same time point in two of the three assays were at 48 h with 0.01 µM and at 72 h with 0.01 µM and 0.1 µM of intermediate **9**. At 48 h, the percentages of untreated cells were 43.11% for living cells, 9.99% for early apoptotic, 33.85% for late apoptotic, and 6.05% for dead cells. After treatment with 0.01 µM of intermediate **9**, the percentages of cells were 42.53% for living cells, 7.75% for early apoptotic, 29.62% for late apoptotic, and 13.14% for dead cells. At 72 h, the percentages of untreated cells were 61.21% for living cells, 4.56% for early apoptotic, 20.64% for late apoptotic, and 9.23% for dead cells. After treatment with 0.01 µM of intermediate **9**, the percentages of cells were 64.78% for living cells, 4.59% for early apoptotic, 12.06% for late apoptotic, and 13.35 for dead cells. After treatment with 0.1 µM of intermediate **9**, the percentages of cells were 61.26% for living cells, 5.63% for early apoptotic, 15.22% for late apoptotic, and 12.76% for dead cells.

On the other hand, final molecule **10** appeared (Table 5) to have significant cytotoxic activity against MDA-MB-231 at 0.1 µM with MTT assay. At 24 h, the percentages of untreated cells were 69.6% for living cells, 3.02% for early apoptotic, 14.24% for late apoptotic, and 2.39% for dead cells. After treatment with 0.1 µM of final compound **10**, the percentages of cells were 56.79% for living cells, 3.16% for early apoptotic, 27.72% for late apoptotic, and 2.52% for dead cells.

## 3. Discussion

The aim of our study was to determine the utility of substituted pyrazine compounds such as SHP2 inhibitors. We built a pharmacophore model using the highly selective SHP2 allosteric inhibitor SHP099–SHP2 complex. A set of three novel compounds was discovered using a structure-based drug design. Compared with the existing inhibitors, the new compounds not only had a similar ability to be bound in the allosteric binding site of SHP2, but also a new hydrogen bond was determined during this binding mode. The modulation of existing inhibitors and designing new inhibitors of SHP2 are really crucial if we consider that SHP2 is a therapeutic target due to the fact of its importance in known oncogenic pathways and emerging role in immuno-oncology. According to the literature, there are amino pyrazines, such as kinase inhibitors [11,16,22,23,24,25], which have different biological data depending on their structures. Hence, in this work, we have focused on allosteric inhibition of SHP2–PTP catalytic sites through small molecules, particularly in amino pyrazine compounds with different novel cyclic amine rings with substitutions. Initially, we started with condensed ring systems. We started with prolines as cyclic amines; through in silico modelling, we came to know that the interactions are as reported in the literature with one new hydrogen bond. Also, we noticed the space available with the ortho position of proline for functionalization. Then, we designed structures with different substitutions at the 2 and 3 positions of the prolines followed by a chain of amides with substitutions.

As result of our efforts, we synthesized three novel pyrazine small molecular prolinamides and sulphonamides as allosteric inhibitors of SHP2 which were determined by structure-based drug design. The new compounds contained the 1-(methylsulfonyl)-4-prolylpiperazine group as a linker. The final compound (**10**) and some of its intermediates (compounds **9**, **21**, **22**) were evaluated for their cytotoxic effect on different human cancer cell lines, and they were examined by four different biological assays. Generally, an increasing dose of the intermediates led to an increasing percentage of apoptosis and necrosis. Concerning the final compound **10**, after treatment with 0.1 µM, the percentages of MDA-MB-231 human breast adenocarcinoma cells were 56.79% for living cells, 3.16% for early apoptotic, 27.72% for late apoptotic, and 2.52% for dead cells. The methodology described to identify allosteric inhibitors also avoided the drug discovery challenges related to the polarity and homology of the orthosteric binding pocket of SHP2. Finally, the compounds **18** and **19** will be evaluated for their cytotoxic effect in different cancer cell lines, and the compound **10** will be modified in order to achieve a better effect, as the results we presented in this work encourage us to further modify our structures.

## 4. Materials and Methods

### 4.1. Small Molecule Docking

The molecular docking studies on SHP2 were performed using ICM version 3.8-7c (Molsoft LLC, San Diego, CA, USA) on a 2.0 GHz Intel Xeon Gold 6138 processor. The X-ray structure of SHP2 (PDB ID: 5EHR) included two copies of the protein, and chain A was chosen as the receptor for docking. The protein was prepared for docking through ICM’s automated protocol. This process includes: the global optimization of hydrogens, the optimization of the orientation of His, Pro, Asn, Gln, and Cys residues, and the deletion of water molecules that are not tightly bound to the protein structure. The binding pocket was defined around the co-crystallized ligand, SHP099, by creating a box around the ligand with a margin of 3. The box defines the potential energy maps and the selected margin value was enough, in this case, to encompass the whole binding site. The co-crystallized ligand, SHP099, was removed before docking. The chosen docking approach involved a rigid receptor and fully flexible ligands. A table of compounds was imported into ICM and docked against the allosteric binding pocket of SHP2 with an effort of 2. The effort value (ranges from 1 to 10) determines the “thoroughness” which relates to the length of the simulation’s sampling time. Conformational sampling uses the Monte Carlo minimization procedure, and the scoring is according to the ICM scoring function [26,27,28]. A docking score was assigned to each docked compound and used to assess the predicted interaction.

### 4.2. Chemistry

#### 4.2.1. General Information

One milligram of synthesized compound was dissolved in 1 mL of a methanol/water mixture (90/10), filtered through a 0.45 μm filter, and inserted in an amber glass vial. The LC/MS analysis (1260 Infinity Series HPLC, 6120 Quadrupole MSD, Agilent Technologies Inc., Richardson, TX, USA) was carried out with the use of a reverse phase column, Zorbax RX-C8 (5 µm, 250 mm × 4.6 mm, Agilent Technologies, Santa Clara, CA, USA) maintained at 40 °C. Chromatograms were integrated and analyzed using OpenLAB Chemstation (version M8301AA, Revision C.01.07 Agilent Technologies Inc., Richardson, TX, USA). The solvent system used was: (A) aqueous solution of 0.1% ammonium acetate and (B) solution of 0.1% ammonium acetate in methanol. The separation was carried out at a 0.4 mL/min flow rate with isocratic elution of 10% solvent A and 90% solvent B for 20 min. The injection volume was 10 µL and ultraviolet (UV) detection was carried out at 278, 280, and 325 nm. Mass spectrometry analysis was performed with a 6120 Series Quadrupole System (Agilent Technologies, Santa Clara, CA, USA) equipped with an electrospray ionization source (ESI) operating in negative mode by scan analysis with a 100–1000 *m/z* scan range. Proton and carbon nuclear magnetic resonance (^1^H and ^13^C-NMR) spectra were recorded with dimethylsulfoxide-d^6^ (in DMSO-d^6^) on the following instrument: Bruker (^1^H at 400 MHz and ^13^C-NMR at 100 MHz, Milano, Italy).

#### 4.2.2. Experimental Procedures and Analytical Data

All reactions were carried out under N_2_ atmosphere unless specified.

*Methyl (6-amino-5-(2,3-dichlorophenyl)pyrazin-2-yl)prolylprolinate hydrochloride* (**10**): To a clean dry round-bottom flask, which was purged with nitrogen to remove water traces and other particles, 3-bromo-6-chloropyrazin-2-amine (1.0 eq) was added and dissolved in dry THF (10 volumes). Then, (2,3-dichlorophenyl) boronic acid (1.3 to 1.5 eq) was added and stirred for 10 min under inert atmosphere. The reaction mass was degassed using a nitrogen inlet and vacuum outlet for 15 min. Tetrakis (triphenylphosphine) palladium (0) (0.1 eq) was added and stirred for 15 min. After the addition of a solution of 2 M Na_2_CO_3_, the reaction mixture was slowly heated to reflux. The process of the reaction was evaluated by TLC and LCMS. After the solution was stirred for 4 h, it was cooled to RT. Ethyl acetate and water were added, and the reaction mixture was stirred for another 10 min. The organic layer was separated, and the aqueous layer was extracted with ethyl acetate. The combined organic layer was washed with brine and dried over sodium sulfate. After evaporation of the solvent, the crude product was purified by flash chromatography (0% to 50% gradient of ethyl acetate/*n*-hexane) to get 6-chloro-3-(2,3-dichlorophenyl)pyrazin-2-amine (**3**) as a yellow crystalline solid. MS *m/z* 274.10/276.11 (M + H)^+^.

To a cooled solution (0 °C) of 6-chloro-3-(2,3-dichlorophenyl)pyrazin-2-amine (**3**) (1.0 eq) in DCM (10 volumes), triethyl amine (2.5 eq) was added and stirred for 10 min. Then, Boc anhydride (1.5 eq) and DMAP (0.1 eq) were slowly added and stirred for 2 h at room temperature. The reaction mixture was diluted with DCM and washed with water and brine solution. The organic layer was dried over sodium sulphate, filtered, and concentrated to get crude compound as a solid. The crude solid was purified by flash chromatography (0% to 15% ethyl acetate/*n*-hexane) to get di-*tert*-butyl- (6-chloro-3-(2,3-dichlorophenyl)pyrazin-2-yl)carbamate (**4**) as a white amorphous solid. MS *m/z* 374.1 (ES-, -100 PEAK).

To a solution of di-*tert*-butyl-(6-chloro-3-(2,3-dichlorophenyl)pyrazin-2-yl)carbamate (1.0 eq) in DMSO (10 volumes) was added cesium carbonate (2.5 eq) and stirred for 10 min. Then proline methyl ester (**5**) (1.5 eq) was slowly added and stirred for another 8 h at 85 °C. The reaction mixture was diluted with ethyl acetate and washed with water and brine solution. The organic layer was dried over sodium sulphate, filtered, and concentrated to get crude compound as a solid. The crude solid was purified by flash chromatography (0% to 50% ethyl acetate/*n*-hexane) to get (6-((di-*tert*-butoxycarbonyl)amino)-5-(2,3-dichlorophenyl)pyrazin-2-yl)prolinate (**6**) as a white solid. MS *m/z* 567.24 (M + H)^+^.

To a cold solution (0 °C) of (6-((di-*tert*-butoxycarbonyl) amino)-5-(2,3-dichlorophenyl)pyrazin-2-yl)prolinate (**6**) (1.0 eq) in THF (10 volumes), methanol (5 volumes) and “LiOH·H_2_O (2.0 eq.) were added were stirred for 10 min. The reaction mixture was warmed to room temperature and stirred overnight. After the completion of the reaction, the solvents were evaporated. Water was added to the crude mass acidified with 6 N HCl until pH = 4. The aqueous layer was extracted with ethyl acetate. The combined organic layer was washed with brine and dried over sodium sulfate, filtered, and concentrated to get crude compound as a solid. The crude mass was washed with ether and dried to get (6-((di-*tert*-butoxycarbonyl) - amino)-5-(2,3-dichlorophenyl)pyrazin-2-yl)proline (**7**) as a white solid. MS *m/z* 551.13/553.11 (M − H)^−^.

To a clean dry round-bottom flask, which was purged with nitrogen to remove water traces and other particles, a solution of (6-((di-*tert*-butoxycarbonyl)amino)-5-(2,3-dichlorophenyl)pyrazin-2-yl) proline (1.0 eq) was added in DMF(10 volumes). Then L-proline methyl ester (**5**) (2.0 eq) in DMF was added and stirred for 10 min. Finally, HATU (2.0eq) and DIPEA (3.0eq) were added and stirred overnight. The reaction mixture was diluted with ethyl acetate and washed with water and brine solution. The organic layer was dried over sodium sulphate, filtered, and concentrated to get crude compound as a solid. The crude solid was purified by flash chromatography (0% to 5% MeOH/DCM) to get methyl (6-((di-*tert*-butoxycarbonyl)amino)-5-(2,3-dichlorophenyl)pyrazin-2-yl)prolylprolinate (**8**) as a colorless liquid.

To a cooled solution (0 °C) of (6-((di*tert*-butoxycarbonyl)amino)-5-(2,3-dichlorophenyl)pyrazin-2-yl)prolylprolinate (**8**) (1.0 eq) in MeOH, 4M HCl was added in methanol (10 volumes) and stirred for 10 min. The reaction mixture was warmed to room temperature and stirred overnight. The reaction mixture was distilled with methanol and dried to get crude mass. The crude product was washed with ether and dried to get methyl (6-amino-5-(2,3-dichlorophenyl)pyrazin-2-yl)prolylprolinate hydrochloride (**9**) as a yellow solid MS *m/z*: 502,11 (M + H)^+ 1^H-NMR (400 MHz, DMSO-d^6^) δ ppm 7.68 (d, *J* = 7.2 Hz, 1H) 7.45 (t, *J* = 7.6 Hz, 1H) 7.36 (d, *J* = 7.2 Hz, 1H) 7.17 (s, 1H) 4.76 (d, *J* = 8 Hz, 1H) 4.34–4.30 (m, 2H) 3.92–3.88 (m, 2H) 3.61–3.50 (m, 4H) 2.29–2.16 (m, 2H) 2.03–1.95 (m, 4H) 1.88–1.81(m, 1H).

*(1-((1-(6-amino-5-(2,3-dichlorophenyl)pyrazin-2-yl)pyrrolidin-2-yl)methyl)pyrrolidin-2-yl)methanol* (**10**): All the intermediates from **1** to **9** were prepared following the procedures above. To a clean dry round-bottom flask, the nitrogen was purged to remove water traces and other particles and charged with methyl (6-amino-5-(2,3-dichlorophenyl)pyrazin-2-yl)prolylprolinate hydrochloride (**9**) dissolved in dry THF. The solution was cooled to 0 °C, added 1M LAH in THF (4.0 eq), and stirred for 10 min. The reaction mixture was warmed to room temperature and was stirred overnight. The reaction was quenched with 1 N aqueous NaOH at 0 °C. Ethyl acetate was added to the mixture and stirred for 15 min and then filtered on a celite pad. The filtrate was concentrated and dried to get crude product. The crude mass was purified by flash chromatography (0 to 7% MeOH/DCM) to get (1-((1-(6-amino-5-(2,3-dichlorophenyl)pyrazin-2-yl)pyrrolidin-2-yl)methyl)pyrrolidin-2-yl)methanol as a yellow solid (**18**). MS *m/z*: 422.15 (M + H)^+ 1^H-NMR (400 MHz, DMSO-d^6^) δ ppm 7.62 (d, *J* = 4.8Hz, 1H) 7.42(dd, *J* = 8Hz, 1H) 7.30(d, *J* = 6Hz, 1H) 7.19 (s, 1H) 5.69(s, 1H) 4.36(t, *J* = 5.2Hz, 1H) 4.08(br, 1H) 3.55(br, 2H) 3.16(br, 2H) 2.60(m, 1H) 2.40(br, 1H) 2.08–2.03(m, 1H) 1.84–1.77(m, 4H) 1.69–1.67(m, 2H) 1.67–1.51(m, 1H) 1.23(s, 1H).

(*1-(6-amino-5-(2,3-dichlorophenyl)pyrazin2yl)pyrrolidin2yl)(4-((trifluoromethyl)sulfonyl)piperazin-1-yl)methanone hydrochloride* (**19**): To a stirred solution of *tert*-butyl-piperazine-1-carboxylate (**11**) in DCM (10 volumes) hunig’s base (2.5 eq) was added and stirred for 10 min. The solution was cooled to −50 °C. Then trifluoromethane sulphonic anhydride (1.5 eq) was slowly added and stirred for 1 hr at −50 °C. The reaction mixture was diluted with DCM and washed with water and brine solution. The organic layer was dried over sodium sulphate, filtered, and concentrated to get crude compound as a solid. The crude solid was purified by flash chromatography to get tert-butyl 4-((trifluoromethyl)sulfonyl)piperazine-1-carboxylate (**13**) as an off-white solid. MS *m/z*: 343.30 (M + 23)^+^, 241.30 (M − 100)^+^.

To a cold stirred solution (at 0 °C) of *tert*-butyl-4-((trifluoromethyl)sulfonyl) piperazine-1-carboxylate (**13**) in MeOH, 4 M methanolic HCl (10 volumes) was slowly added and stirred overnight at room temperature. The reaction mass was distilled with methanol and dried to get crude mass. The reaction mixture was washed with ether and dried to get 1-((trifluoromethyl)sulfonyl)piperazine hydrochloride (**15**) as a light brown solid. MS *m/z*: 219.11 (M + H)^+^.

To a clean dry round-bottom flask, which was purged with nitrogen to remove water traces and other particles, a solution of (6-((Di*tert*-butoxycarbonyl)amino)-5-(2,3-dichlorophenyl)pyrazin-2-yl)proline (1.0 eq) (**7**) was charged in DMF (10 volumes). Then, 1-((trifluoromethyl)sulfonyl)piperazine hydrochloride (**15**) (2.0 eq) in DMF was added and stirred for 10 min. Finally, HATU (2.0 eq) and DIPEA (3.0 eq) were added and stirred overnight. The reaction mixture was diluted with ethyl acetate and washed with water and brine solution. The organic layer was dried over sodium sulphate, filtered, and concentrated to get crude compound as a solid. The crude solid was purified by flash chromatography (0% to 5% MeOH/DCM) to get di-*tert*-butyl- (3-(2,3-dichlorophenyl)-6-(2-(4-((trifluoromethyl)sulfonyl)piperazine-1-carbonyl)pyrrolidin-1-yl)pyrazin-2-yl)carbamate (**17**) as a white solid. MS *m/z*: 753.23 (M + H)^+^.

To a cooled (at 0 °C) stirred solution of Di-*tert*-butyl (3-(2,3-dichlorophenyl)-6-(2-(4-((trifluoromethyl)sulfonyl)piperazine-1-carbonyl)pyrrolidin-1-yl)pyrazin-2-yl)carbamate (**17**) in MeOH, 4 M methanolic HCl (10 volumes) was slowly added and stirred for overnight at room temperature. The reaction mass was distilled with methanol and dried to get crude mass. The reaction mass was washed with ether and dried to get (1-(6-amino-5-(2,3-dichlorophenyl)pyrazine-2-yl)-pyrrolidin-2-yl)-(4-((trifluoromethyl)sulfonyl)-piperazin-1-yl)methanone hydrochloride (**19**) as a yellow solid. MS *m/z*: 555.15 (M + H)^+^. ^1^H-NMR (400 MHz, DMSO-d^6^) δ ppm 7.72 (d, *J* = 4Hz, 1H) 7.47 (dd, *J* = 7.6Hz, 1H) 7.40 (d, *J* = 8Hz, 1H) 7.23 (s, 1H) 4.97(d, *J* = 7.2Hz, 1H) 3.65 (br, 8H) 3.45 (t, *J* = 16Hz, 1H) 2.26 (m, 1H) 2.01(m, 3H) 1.66 (t, *J* = 8Hz, 1H).

*(1-(6-amino-5-(2,3-dichlorophenyl)pyrazin-2-yl)pyrrolidin-2-yl)(4(methylsulfonyl)piperazin-1-yl)methanone hydrochloride* (**18**): To a stirred solution of tert-butyl piperazine-1-carboxylate (**11**) in DCM (10 Volumes), triethyl amine (2.5 eq) was added and stirred for 10 min. The resulting solution was cooled to 0 °C and methane sulphonyl chloride (1.5 eq) was slowly added and stirred overnight at room temperature. The reaction mass was diluted with DCM and washed with water and brine solution. The reaction mixture was diluted with DCM and washed with water and brine solution. The organic layer dried over sodium sulphate, filtered, and concentrated to get crude compound as solid. Crude solid was purified by flash chromatography (0% to 30% ethyl acetate/*n*-hexane) to get tert-butyl 4-(methylsulfonyl) piperazine-1-carboxylate (**12**) as a pale brown solid. MS *m/z*: 265.19 (M + H)^+^.

To a cold stirred solution (at 0 °C) of *tert*-butyl-4-(methylsulfonyl) piperazine-1-carboxylate (**12**) in MeOH, 4 M methanolic HCl (10 volumes) was slowly added and stirred overnight at room temperature. The reaction mass was distilled with methanol and dried to get a crude mass. The reaction mass was washed with ether and dried to get 1-(methylsulfonyl)piperazine hydrochloride (**14**) as a light brown oil. MS *m/z*: 165.15 (M + H)^+^.

To a clean dry round-bottom flask, which was purged with nitrogen to remove water traces and other particles, (6-((Di-*tert*-butoxycarbonyl)amino)-5-(2,3-dichlorophenyl)pyrazin-2-yl)proline (**7**) was charged, dissolved in DMF (10Volumes) and 1-(methylsulfonyl)piperazine hydrochloride (**14**) (2.0 eq) in DMF and the resulting mixture was stirred for 10 min. Finally, HATU (2.0 eq) and DIPEA (3.0 eq) were added and stirred overnight. The reaction mass was diluted with ethyl acetate, washed with water and brine solution. The reaction mixture was diluted with ethyl acetate and was washed with water and brine solution. The organic layer was dried over sodium sulphate, filtered, and concentrated to get crude compound as a solid. The crude mass was purified with flash chromatography (0% to 5% MeOH/DCM) to get di-tert-butyl(3-(2,3-dichlorophenyl)-6-(2-(4-(methylsulfonyl) piperazine-1-carbonyl)pyrrolidin-1-yl)pyrazin-2-yl)carbamate (**16**) as a white solid. MS *m/z*: 699.26, 701.23 (M + H)^+^.

To a cold stirred solution (at 0 °C) of di-*tert*-butyl (3-(2,3-dichlorophenyl)-6-(2-(4-(methylsulfonyl)piperazine-1-carbonyl)pyrrolidin-1-yl)pyrazin-2-yl)carbamate (**16**) in MeOH, 4 M methanolic HCl (10 volumes) was slowly added and stirred overnight at room temperature. The reaction mass was distilled with methanol and dried to get crude mass. The reaction mass was washed with ether and dried to get (1-(6-amino-5-(2,3-dichlorophenyl)pyrazin-2-yl)pyrrolidin-2-yl)(4-(methylsulfonyl)piperazin-1-yl)methanone hydrochloride (**18**) as a yellow solid. MS *m/z*: 499.16, 501.16 (M + H)^+^. ^1^H-NMR (400 MHz, DMSO-d^6^) δ ppm 8.50 (br, 2H) 7.68 (d, *J* = 8.0Hz, 1H) 7.45–7.35 (m, 2H) 7.17 (s, 1H) 4.98 (d, *J* = 7.6Hz, 1H) 3.78 (br, 2H) 3.51 (br, 2H) 3.14 (br, 2H) 2.88 (s, 3H) 2.52–2.49 (m, 5H).

*(3-(2,3-dichlorophenyl)-6-(2-(hydroxymethyl)pyrrolidin-1-yl)pyrazin-2-yl)carbamate* (**21**): All the intermediates from **1** to **4** were prepared as described above. To a clean dry round-bottom flask, which was purged with nitrogen to remove water traces and other particles, Di-*tert*-butyl- (6-chloro-3-(2,3-dichlorophenyl)pyrazin-2-yl)carbamate (**4**) was charged, dissolved in DMSO (10 volumes) and stirred for 10 min. Then cesium carbonate (2.5 eq) was added and stirred for 10 min. Prolinol (1.5 eq) was slowly added and the reaction mixture was stirred for 8 h at 85 °C. The reaction mixture was diluted with ethyl acetate and washed with water and brine solution. The organic layer was dried over sodium sulphate, filtered, and concentrated to get crude compound as a solid. The crude solid was purified by flash chromatography (0% to 60% ethyl acetate/*n*-hexane) to get Di-*tert*-butyl-(3-(2,3-dichlorophenyl)-6-(2-(hydroxymethyl)pyrrolidin-1-yl)pyrazin-2-yl)carbamate as a colorless solid (**21**). ^1^H-NMR (400 MHz, DMSO-d^6^) δ ppm 8.1 (s, 1H) 7.67 (d, *J* = 8Hz, 1H) 7.43 (t, *J* = 7.6Hz, 1H) 7.29 (d, *J* = 8Hz, 1H) 4.84 (s, 1H) 4.1 (s, 1H) 3.58–3.54 (m, 4H) 2.03–1.95 (m, 4H) 1.28 (s, 9H).

To a cold stirred solution (at 0 °C) of Di-*tert*-butyl-(3-(2,3-dichlorophenyl)-6-(2-(hydroxymethyl)pyrrolidin-1-yl)pyrazin-2-yl)carbamate (**21**) in MeOH, 4 M methanolic HCl (10 Volumes) was slowly added and stirred overnight at room temperature. The reaction mass was distilled with methanol and dried to get crude mass. The reaction mass was washed with ether and dried to get (1-(6-amino-5-(2,3-dichlorophenyl)pyrazin-2-yl)pyrrolidin-2-yl)methanol hydrochloride (**22**) as a yellow solid. ^1^H-NMR (400 MHz, DMSO-d^6^) δ ppm 7.76 (d, *J* = 7.6Hz, 1H) 7.49–7.43 (m, 2H) 7.39 (s, 1H) 4.12(s, 1H) 3.54–3.39 (m, 5H) 2.01–1.92 (m, 4H).

### 4.3. Cell Culture

Commercial cancer cell lines were purchased from ECACC (European Collection of Authenticated Cell Cultures) (Salisbury, UK). Cells were cultured in a humidified cell incubator at 37 °C and 5% CO_2_ and passaged when cells reached 80% confluence. The MCF7 human breast adenocarcinoma cells (luminal type) (ECACC 86012803) were cultured in RPMI 1640 supplemented with 10% FBS, 2% l-Glutamine and 2% NEAA. MDA-MB-231 human breast adenocarcinoma cells (triple negative) (ECACC 92020424) were cultured in RPMI 1640 supplemented with 15% FBS and 2% l-Glutamine. HCT116 human colorectal carcinoma cells (ECACC 91091005) were cultured in DMEM supplemented with 10% FBS and 2% l-Glutamine. The MOR human lung adenocarcinoma cells (ECACC 84112312) were cultured in RPMI 1640 supplemented with 10% FBS and 2% l-Glutamine. RPMI 1640 Catalog#R0883 and l-Glutamine Catalog#G7513-100ML were obtained from Sigma–Aldrich, Darmstadt, Germany, and FBS Catalog#FB-1001/500 was obtained from BioSera, Nuaille, France. The MEM non-essential amino acids (NEAA) Catalog #M7145-100ML and DMEM (Dulbecco’s modified Eagle’s medium) Catalog #D5546 were purchased from Sigma–Aldrich, Darmstadt, Germany.

### 4.4. Cell Viability Analysis

Viability was measured with MTT for cell-metabolism activity, with SRB and CVE for cellular protein content. The viable cells were seeded at a density of 2 × 10^4^ (200 µL/well) in 96 well plates and incubated at 37 °C and 5% CO_2_ for 24 h to form a cell monolayer. After 24 h of incubation, supernatant on the monolayer was aspirated and 200 µL of medium and varying concentrations of the natural substances were added and incubated for 24, 48, and 72 h time points.

After the specific time points, 20 µL of 5 mg/mL MTT Catalog #M2128 (Sigma–Aldrich, Darmstadt, Germany) in PBS Catalog #P3813 (Sigma–Aldrich, Darmstadt, Germany) was added to each well and incubated for 3 h at 37 °C and 5% CO_2_. Supernatants were discarded and 100 µL of DMSO Catalog #445103 (Carlo Erbo Reagents, Barcelona, Spain) was added and the plates were incubated for 5 min at 37 °C and 5% CO_2_ to solubilize the formazan crystals. The absorbance was measured at 560 nm, and the reference wavelength was at 605 nm.

For the SRB assay, at the specific time points cells were fixed by adding 100 µL trichloroacetic acid Catalog #91228 (Sigma–Aldrich, Darmstadt, Germany) and with incubation at 4 °C for 1 h. Next, 100 µL of SRB solution Catalog #341738 (Sigma–Aldrich, Darmstadt, Germany) was added in the wells for 30 min at room temperature. After that, supernatant was discarded, cells were rinsed three times with 1% glacial acetic acid Catalog #1.00063.1011 (Merck, Darmstadt, Germany) and were left to air dry. Finally, the dye was solubilized with 200 µL of 10 mM Tris-base pH 10.5 Catalog #T6791 (Sigma–Aldrich, Darmstadt, Germany) and absorbance was measured at 570 nm and the reference wavelength was at 605 nm.

For the CVE assay, at the specific time points, supernatant was removed and cells were fixed with 100 µL 10% formalin for 20 min. Formalin was discarded and cells were left to air dry. Next, cells were dyed with 100 µL of 0.25% aqueous crystal violet solution Catalog #HT901 (Sigma–Aldrich, Darmstadt, Germany) and left for 10 min at room temperature. Then, the supernatant was discarded, and cells were rinsed twice with 100 µL WFI (water for injection) and were left to air dry. The dye was solubilized with 100 µL of 33% glacial acetic acid Catalog #1.00063.1011 (Merck, Darmstadt, Germany). Absorbance was measured at 570 nm and the reference wavelength was at 605 nm.

### 4.5. Apoptosis Assessment

Apoptosis detection was conducted with flow cytometry to determine the membrane phospholipid phosphatidylserine exposed apoptotic cells by Annexin V-PE and 7-AAD double staining. The PE Annexin V Apoptosis Detection Kit I Catalog #559763 (BD Biosciences, San Jose, CA, USA) was used for carrying out the experiments by following the manufacturer’s instructions. Samples were analyzed on FACScan Calibur (Becton Dickinson, San Jose, CA, USA) and FSC 6 Express software.

## 5. Conclusions

In this work we established that, with the effectiveness of structure-based drug design, we were able to accomplish the novel 1-(methylsulfonyl)-4-prolylpiperazine group as a linker in pyrazine amine compounds. These structures have shown a receivable anti-cancerous activity.

## Figures and Tables

**Table 1:** Cytotoxicity (%) results of intermediate **9** against human cancer cell lines according to viability assays (i.e., MTT, SRB, CVE). There were no statistically significant effects against the MDA-MB-231 cell line at any assay and at any time point.

Intermediate 9	24h			48h			72h		
	MTT	SRB	CVE	MTT	SRB	CVE	MTT	SRB	CVE
**MCF7**	-	-	0.1 µM (17%)	0.001 µM (27%)	-	0.001 µM (9%)	0.001 µM (12%), 0.01 µM (11%), 1 µM (9%)	0.1 µM (7%), 1 µM (10%)	
**HCT116**	0.01 µM (18%), 0.1 µM (16%)	-	-	-	-	-	-		-
**MOR**	0.001 µM (20%), 0.1 µM (13%)	-	-	0.001 µM (19%), 0.01 µM (16%)	0.01 µM (13%)	-	0.001 µM (30%), 0.01 µM (31%)	0.01 µM (25%)	0.1 µM (9%)

**Table 2:** Cytotoxicity (%) results of intermediate **10** against human cancer cell lines according to viability assays (i.e., MTT, SRB, CVE).

Intermediate 10	24 h			48 h			72 h		
	MTT	SRB	CVE	MTT	SRB	CVE	MTT	SRB	CVE
**MCF7**	0.001 µM (13%), 0.1 µM (20%)	-	1 µM (10%)	-	-	0.01 µM (6%)	-	-	0.001 µM (8%), 0.01 µM (11%), 0.1 µM (15%), 1 µM (8%)
**MDA-MB-231**	0.01 µM (16%), 0.1 µM (30%)	-	-	-	-	-	0.1 µM (13%)	-	-
**HCT116**	0.001 µM (19%), 0.01 µM (14%), 0.1 µM (32%)	-	-	-	-	-	0.01 µM (25%), 0.1 µM (22%)	-	-
**MOR**	0.001 µM (17%), 0.01 µM (28%), 0.1 µM (27%)	-	-	0.01 µM (31%), 0.1 µM (23%)	1 µM (24%)	-	0.001 µM (25%), 0.01 µM (38%), 0.1 µM (33%)	-	-

Determination of apoptosis by flow cytometry.

**Table 3:** Apoptosis results in MCF7 after the treatment with intermediate **9**.

MCF7	Untreated (48h)	0.001 µM (48 h)	Untreated (72h)	1 µM (72 h)
Living Cells	88.48%	86.53%	78.21%	78.21%
Early Apoptotic	0.41%	0.38%	1.62%	1.51%
Late Apoptotic	1.84%	1.38%	6.45%	5.91%
Dead Cells	1.3%	2.34%	1.52%	2.34%

**Table 4:** Apoptosis results in MCF7 after the treatment with intermediate **9**.

MOR	Untreated	0.01 µM (48 h)	Untreated	0.01 µM (72 h)	Untreated	0.1 µM (72 h)
Living Cells	43.11%	42.53%	61.21%	64.78%	61.21%	61.26%
Early Apoptotic	9.99%	7.75%	4.56%	4.59%	4.56%	5.63%
Late Apoptotic	33.85%	29.62%	20.64%	12.06%	20.64%	15.22%
Dead Cells	6.05%	13.14%	9.23%	13.35%	9.23%	12.76%

**Table 5:** Apoptosis results in MDA-MB-231 after the treatment with final molecule **10**.

MDA-MB-231	Untreated	0.1 µM (24 h)
Living Cells	69.6%	56.79%
Early Apoptotic	3.02%	3.16%
Late Apoptotic	14.24%	27.72%
Dead Cells	2.39%	2.52%
